# Electromagnetic acoustic imaging methods: resolution, signal-to-noise, and image contrast in phantoms

**DOI:** 10.1117/1.JMI.8.6.067001

**Published:** 2021-12-20

**Authors:** Jane F. Emerson, David B. Chang, Stuart McNaughton, Ellen M. Emerson, Stephen A. Cerwin

**Affiliations:** a University of Southern California, Department of Pathology, Los Angeles, California, United States; b Magnetus LLC, Irvine, California, United States; c University of Southern California, Keck School of Medicine, Los Angeles, California, United States; d Southwest Research Institute, Institute Scientist (ret.), San Antonio, Texas, United States

**Keywords:** electromagnetic acoustic imaging, ultrasound, hybrid imaging, conductivity-weighted ultrasound, radio-frequency imaging

## Abstract

**Purpose:** Electromagnetic acoustic imaging (EMAI) is a hybrid imaging technique using radio-frequency irradiation to induce ultrasound (US), providing an US image in which spatial conductivity differences provide image contrast. The method is potentially clinically important in that the added diagnostic parameter has been shown to be useful in cancer detection and vascular space delineation.

**Approach:** We report the development of coil configurations and imaging processing techniques designed to address the low signal-to-noise of EMAI and demonstrate achievable resolution and contrast in phantoms along with EMAI signals in excised animal tissue. Experiment results are compared with theoretical calculations.

**Results:** EMAI signal intensities depend on the square of the ampere-turns in the coil radio frequency coil as predicted theoretically. Resolution is shown to be comparable to conventional US imaging with contrast and signal intensity depending on source conductivity. Optimizing signal-to-noise depends on coil design, orientation of the electromagnetic fields, and coherent processing.

**Conclusions:** Two-dimensional EMAI images are shown to have the expected resolution of conventional US with image contrast dependent on conductivity. Achievable signal-to-noise is sufficient to form potentially clinically useful images.

## 1 Introduction

Electromagnetic acoustic imaging (EMAI) is an imaging technique in which long wavelength radio frequency irradiation (RF) is used to induce alternating electric fields in regions of interest. The induced electric fields then generate first and second harmonic ultrasound (US) by virtue of intrinsic electrical properties in the target.^1^ In contrast to RF imaging techniques that rely on thermoelastic expansion, in EMAI the inductively applied E field across electrical conductivity gradients naturally occurring in tissue results in mechanical stress. The resulting mechanical stress that generates a specific frequency ultrasonic signal depends on the square of the amplitude of the applied E field. The theoretical development and experimental verification of EMAI was previously reported.^1^ The expression for the induced US was shown to be

$$\nabla^{2}P-\frac{1}{V_{s}^{2}}\frac{\partial^{2}P}{\partial t^{2}}=\nabla \cdot [\sigma E\times B+\nabla \cdot \{(\varepsilon_{q}+\sigma /i\omega )E\}E],$$ (1)

where P is the US pressure, $V_{s}$ is the speed of sound, σ is the electrical conductivity, ε is the permittivity, q is the charge, ω is the angular frequency, and electric and magnetic induction fields are represented by E and B, respectively. In the absence of a static B field and with typical values of ε, the significant source for generated US is the third term on the right-hand side of Eq. (1) which is dependent on $E^{2}$ and the gradient of the conductivity. (Note that with long wavelengths the gradient of the electromagnetic field itself is negligible.) The US signal is frequency doubled because the mechanical stress responds to the amplitude of the induced E field and is insensitive to polarity. Thus, there are two US emissions per cycle of the applied RF excitation that are frequency- and phase-locked to the excitation frequency. In EMAI with a time varying E field induced by RF irradiation at frequency f, compressible phantoms with regions of varying conductivity will generate US signals at the specific frequency 2f with signal intensity proportional to the value of conductivity gradients, the induced electric field, E, and the RF frequency. In regions of interest that allow for the buildup of interface surface charges, which would introduce a static E field, 1f signals are also predicted. The resulting image contrast depends on acoustic and electrical properties of the inspected region.

For medical imaging, EMAI offers a means of obtaining conductivity weighted US along with automatic overlay onto conventional US images. The contribution of electrical properties to image contrast adds an important diagnostic parameter as it has been shown to be a distinguishing factor in malignancies^2^^–^^4^ and has the potential to enhance edge detection in blood-filled spaces.^1^^,^^5^ A method similar to EMAI was recently shown to be effective in imaging blood vessels both *in vivo* and *in vitro*.^5^ The RF source employed in those demonstrations used a simple RLC ringing circuit as opposed to those used in previous EMAI experiments and those reported here.

*In-vivo* diagnostics in cancer detection rely on techniques that exploit tissue parameters reflecting the microenvironment(s) characteristic of malignancy. Various imaging methods that leverage tissue acoustic properties, electrical properties, or a combination thereof, are currently in use or proposed and under development. Conventional US with elastography provides diagnostic information based on acoustic properties including density and stiffness.^6^ Electrical or conductivity-based imaging offers additional diagnostic features given that malignant tissue is generally more conductive than benign, in part due to altered vascularity. Electromagnetic imaging (RF or microwave)^7^ and electrical impedance tomography (EIT)^8^ are examples of modalities dependent on electrical properties in tissue. They are limited in resolution by long wavelengths, and in the case of EIT, by nonlinearity and poor depth of penetration.^8^ Hybrid imaging techniques include photoacoustic tomography (PAT)^9^ and EMAI. In PAT, absorption from pulsed optical, RF, or microwave irradiation induces US based on thermal properties rather than directly on electrical parameters.^9^ In EMAI, the specific-wavelength US signal induced by RF illumination depends strictly on electrical properties of tissues. Advantages of the EMAI approach include a depth penetration of the E field that is coil-dependent in a fashion comparable to MRI, image formation parameters consistent with US, the ability to be performed in dual mode with conductivity-weighted US providing automatic image overlay, and therapeutic potential using time-reversed ablative US.

Both signals were demonstrated previously in various phantoms consisting of compartments with distinct values of electrical conductivity. Here we report further development of the imaging modality with demonstration of achievable signal-to-noise in excised tissue phantoms, qualitatively show resolution and image contrast in two-dimensional (2-D) imaging, and expand on modeling with implementation of methods for RF delivery and signal processing in EMAI.

## 2 Methods

### 2.1 Equipment

For all the EMAI experiments, the general equipment configuration consisted of an RF coil assembly, a water tank with US transducer(s), an RF burst transmitter, oscilloscope, and compressible phantoms providing varying regions of conductivity (inclusive of immersion solutions). The system was set up to enable RF transmission with US reception or to operate in pulse-echo mode with US transmission and reception. Water tanks were assembled to house the US transducer and phantom and modified for the particular coil assembly in use.

#### 2.1.1 Burst transmitter

An RF burst transmitter operating up to 30 kW delivered pulses at 1-ms repetition rates with pulse lengths of 3 to 5  μs. Maximizing EMAI pressure calls for higher RF frequency, however as US resolution is inversely proportional to f, and long-term goals call for dual-mode EMAI/US imaging, the value of 5 MHz was selected to fit within conventional US imaging frequency ranges. Maximum coil current was on the order of 200 A peak-to-peak which produced typical coil parameters of 2000 to 3000 ampere-turns (A-T) p-p depending on coil geometry. Digital logic circuitry was used to generate the radiofrequency (RF) burst that was subsequently amplified and used to drive the RF coil(s). The same circuitry generated the in-phase (I) and quadrature (Q) reference signals for the coherent signal processing. The various frequencies at specific phase relationships were derived from the same 40-MHz crystal-controlled master clock to ensure coherence. Digital divider chains were used to create the I and Q signals.

The final amplifier used to drive the coils was a tube type pulse amplifier circuit using a 3CPX1500 ceramic vacuum tube. The various coil configurations were incorporated as part of the plate output circuitry. In the case of the low inductance coils, a simple parallel resonant topology was used. For coils with higher inductance, a split-capacitor step-up configuration was used to maximize coil current. In all cases most of the RF power in the generated pulse was absorbed in the resistor used to set circuit Q. The value for circuit Q required a compromise between the goals of minimizing pulse width (low Q) to improve spatial resolution and maximizing the field (high Q) to increase the EMAI signal. Coupling efficiency between the transmitter and coil was very high since the coil was an integral part of the transmitter itself. Coupling from the coil into the saline bath and phantom was highly variable and dependent on positioning.

#### 2.1.2 RF coils

All RF coils used were lab-developed and designed to maximize the magnetically induced E field in the phantoms. Coils were inductively coupled to a saline bath and not in direct physical contact. Further, mechanical contact between the coil or coil form with the bath was prevented to avoid unwanted US ingress arising from magnetic forces on the coil during high power excitation.

The following is a description of the coil assemblies fabricated along with theoretical predictions of field and EMAI signal optimizations.

##### Coil 1: 20-turn, 76-mm Helmholtz coils

For Helmholtz coils, the approximate expression for the EMAI pressure applied when the conductivity change occurs over a distance that is small compared with the acoustic wavelength of the generated US, as would be the case at boundaries between tissues of different electrical properties, is

$$P_{\mathrm{EMAI}}=\frac{\pi \mu^{2}}{4}f▵\sigma (\mathrm{NI}{)}^{2},$$ (2)

where μ is the magnetic permeability, f is the RF frequency, Δσ is the change in conductivity, and NI is the product of the number of turns in the coil and the current in A-T.

**Fig. 1:**
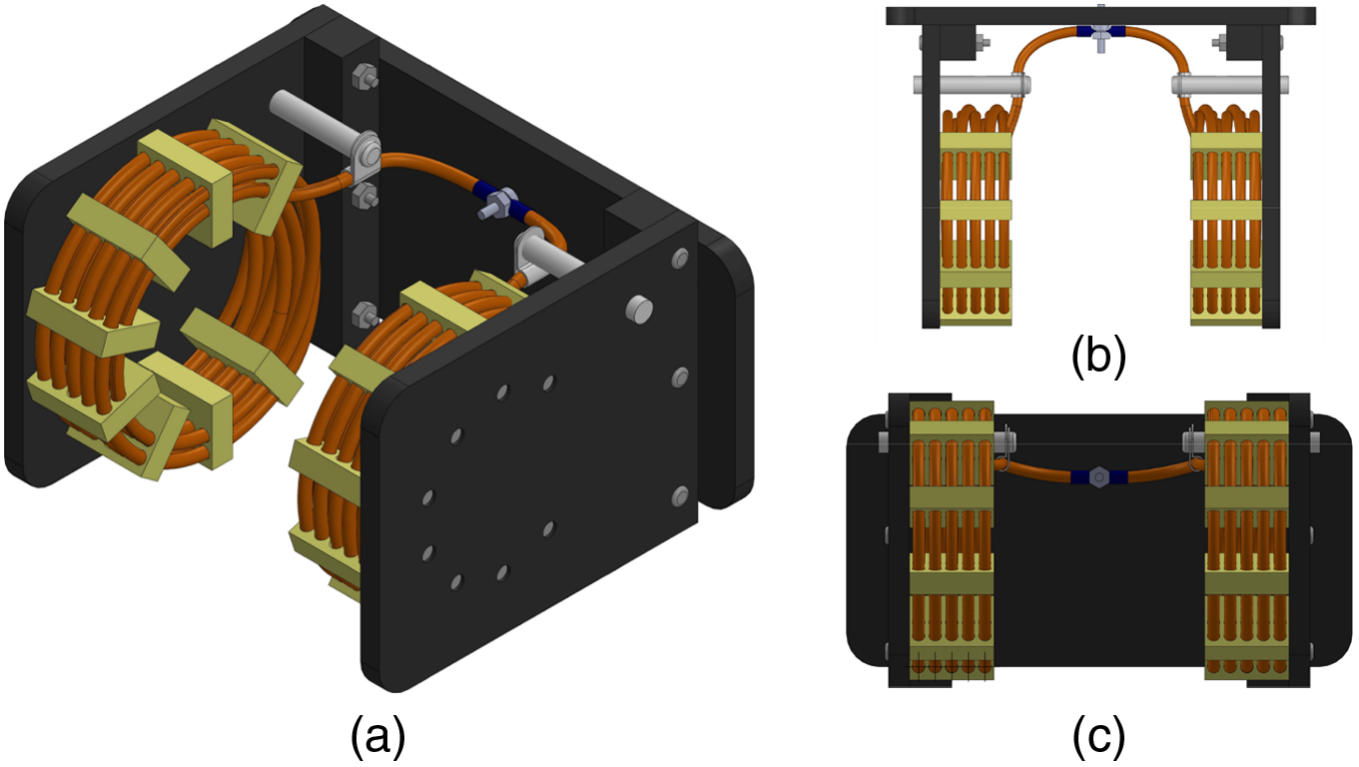
A 76-mm diameter high-voltage Helmholtz coil with 10 turns per side from (a) isometric, (b) top, and (c) front views.

The intent of this coil design (Fig. 1) was to maximize the B field magnitude across the gap. Coil lengths were minimized and the 10 turns on each side were wound in two layers so all turns remained as close to the center as possible. High coil current (>200 A p-p) required innovative design to prevent arcing: high voltage (20 kV) insulated wire was used with generous turn spacing, the winding direction avoided proximity between the hot and cold end, and the two coil halves were connected in parallel instead of the typical series configuration for Helmholtz coils, thereby reducing the reactance and voltage across the coil by a factor of four.

##### Coil 2: Helmholtz coils with ferrite rods

**Fig. 2:**
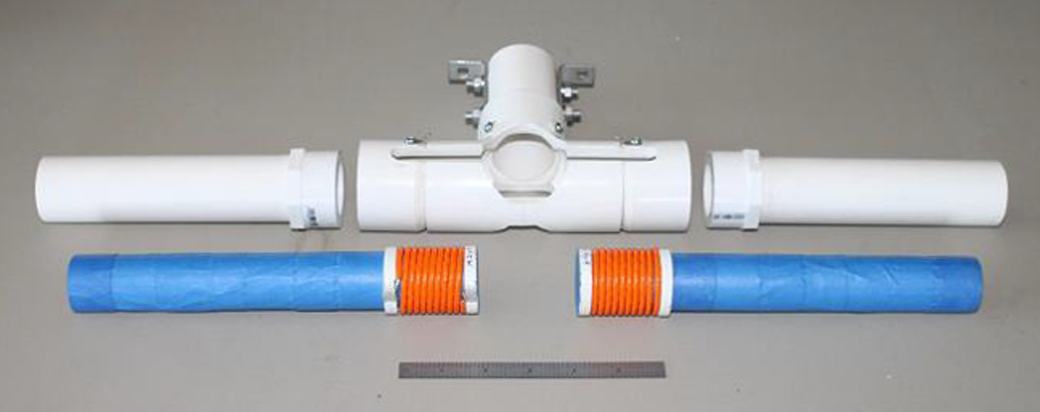
Configuration of 20-turn Helmholtz coils (28 mm) with ferrites rods (blue). PVC pipes (white) were configured to house the coils with a water tank inserted into the center cylinder.

Since the magnitude of EMAI signals for Helmholtz coils depends on $B^{2}$, the addition of ferrites to the coil assembly was predicted to increase SNR. With ferrite rods, the effective NI is raised by a factor of $\mu /\mu_{\mathrm{eff}}$ where μ is the permeability without the rods and $\mu_{\mathrm{eff}}$ is the permeability with the rods. Two ferrite loaded 10-turn Helmholtz pairs were constructed with the configuration shown in Fig. 2 with either a 28 mm diameter or 70 mm diameter. In each case, the ferrite rod extended beyond the coils (total length 24.1 cm). The direction of magnetic field was transverse to the water tube containing the ultrasonic transducer at the bottom. Therefore, the B field direction was perpendicular to the direction of US detection.

##### Coil 3: Modified Helmholtz coils

**Fig. 3:**
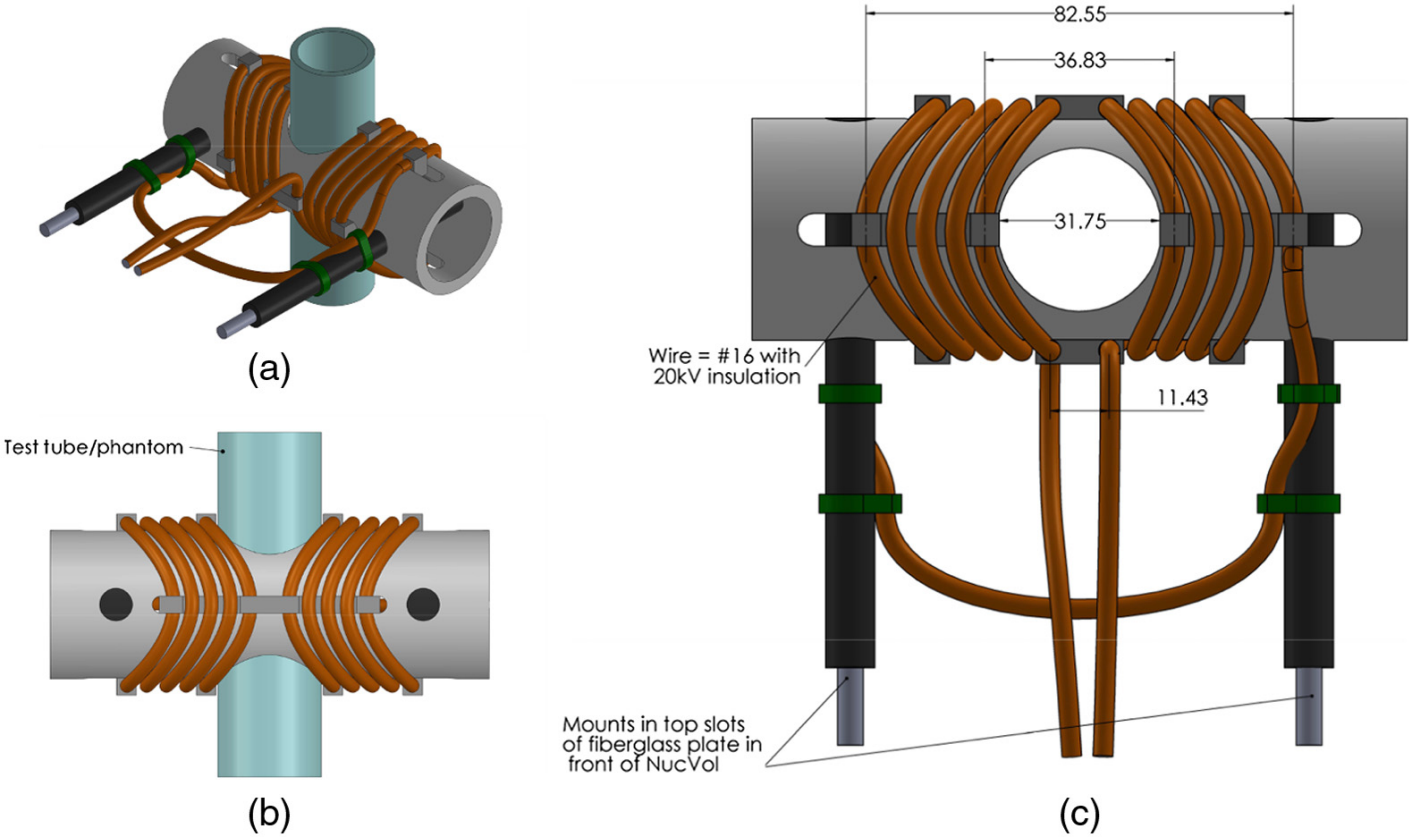
The modified 10-turn Helmholtz coil pair from (a) isometric, (b) back, and (c) top views. Units of measurement are millimeters.

A modified 10-turn Helmholtz pair with a diameter of 47 mm and a 32 mm gap (Fig. 3) was constructed to maximize both B and E fields in the sample volume. The coils were wound such that the hot and cold ends of the coil face each other, and the current carrying conductors are as close to the phantom as possible while allowing for positioning within the water tank. This geometry placed the maximum possible electrostatic field across the inspection volume. The resulting E field was measured at 32 kV across the gap using a capacitive E-field probe. With this configuration, the largest stress on a conductive phantom in nonconductive water will result from the electrostatic E field due to the gap rather than the case in which the E field was due solely to the induction field. The latter follows in the setting of saline shielding the electrostatic field. In this case, both the E and B fields are transverse to the water tube and to the ultrasonic transducer at the bottom.

##### Coil 4: Solenoid

**Fig. 4:**
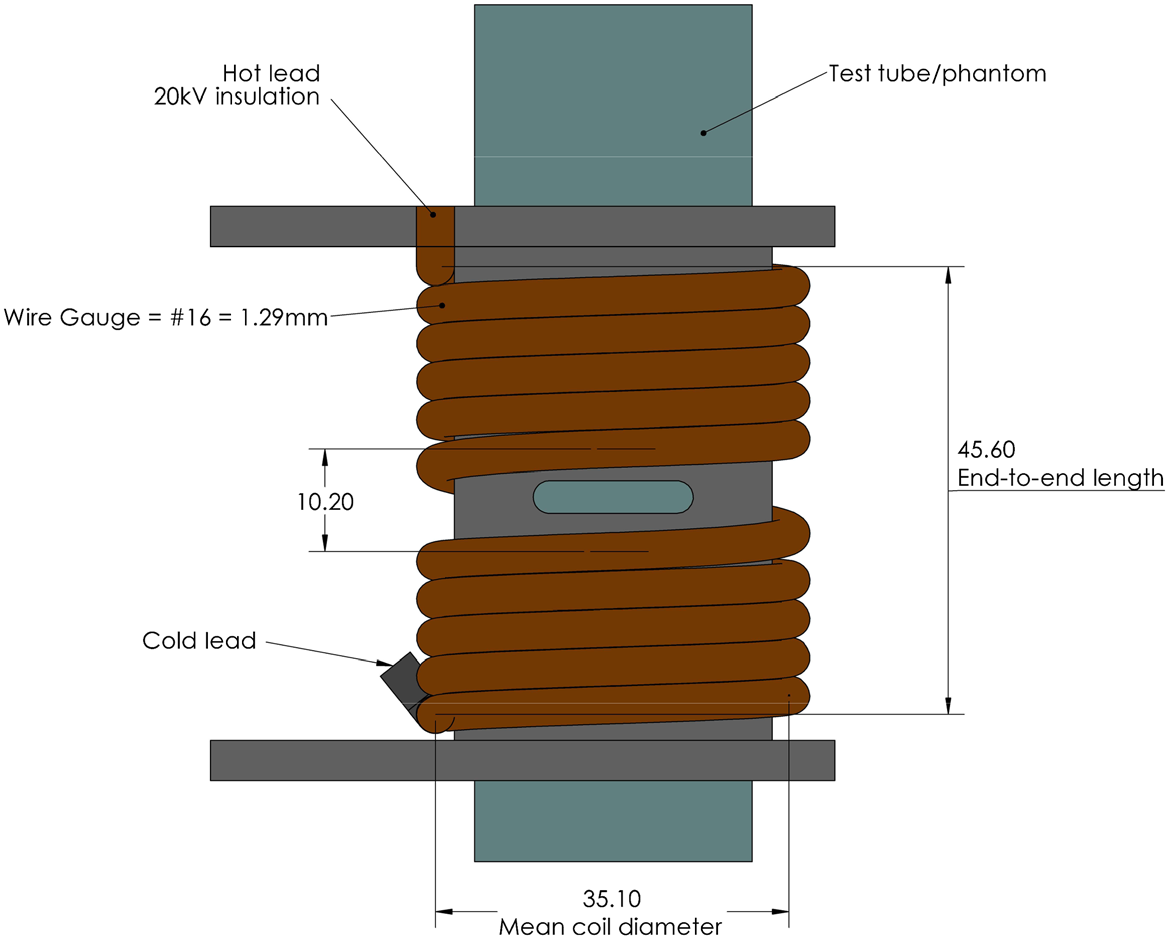
Modified 10-turn solenoid with gap for visualizing phantom placement. Units of measurement are millimeters.

To investigate the effect of a circumferential current surrounding the phantom, a 10-turn solenoid was constructed as shown in Fig. 4. The coil length was 45.6 mm with an outer diameter of 38.6 mm. The nominal turn spacing was 3.8 mm with 10.2 mm gap for phantom visualization. The presence of this gap separating the upper and lower turns defines this configuration as a modified series-connected Helmholtz coil. The intent of this coil configuration was to minimize the phantom volume thereby maximizing flux density. Because the transducer is at the bottom of the water tube, this configuration coaxially aligned the directions of US propagation and the B field.

##### Coil 5: Toroid with ferrite sleeves

**Fig. 5:**
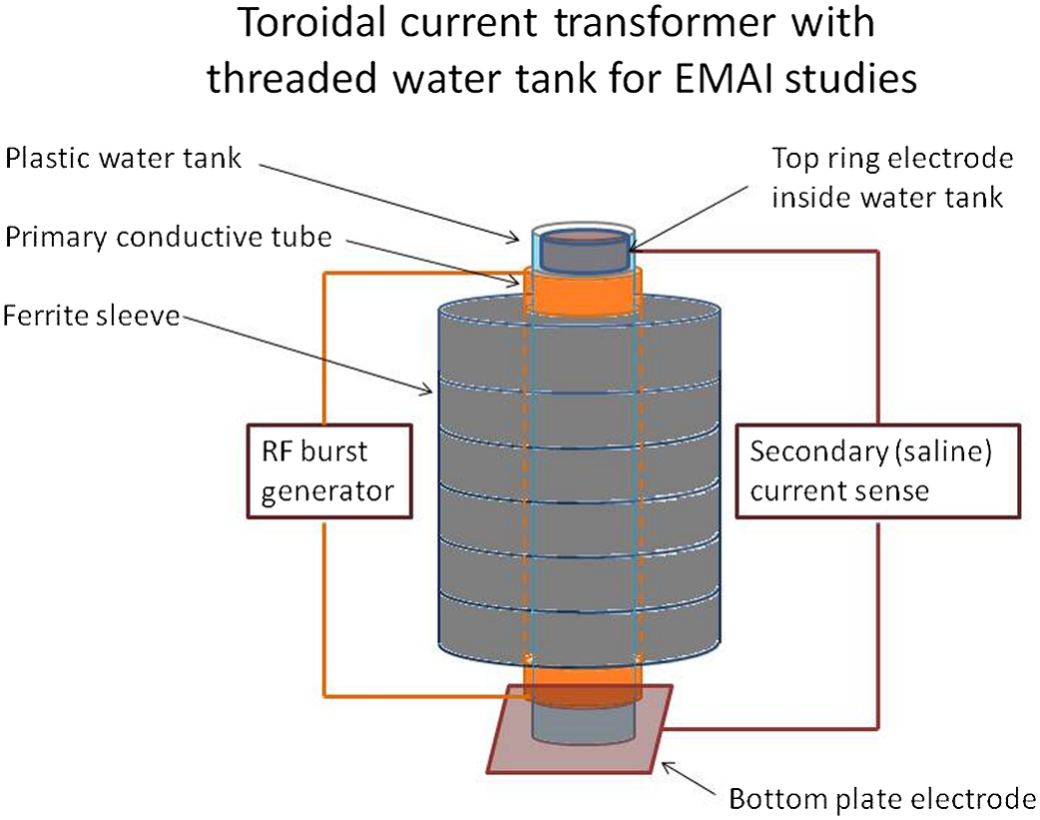
Transmission line transformer with ferrite sleeves designed to improve efficiency of inducing large currents through the phantom.

An axial current configuration was tested with the coil as shown in Fig. 5. Ferrite sleeves were included to raise the effective NI as discussed above. This configuration is essentially that of ferrite-loaded transmission line transformer. The primary “winding” was a conductive sleeve placed on the outside of a nonconductive plastic water tube. The secondary “winding” was the conductive saline solution inside the tube. This geometry tightly couples both the electric and magnetic fields from primary to secondary. The ferrite rings around the primary sleeve increased inductance to a value that could be resonated at the transmitter frequency with an external capacitor. The resulting tuned circuit was coupled to the high-power RF burst transmitter. Circumferential electrodes placed around the inside of the water tube provided electrical connection to the conductive saline column. This enabled the connection of an external circuit for measuring induced current and to provide an optional closed-loop conductive path for sensing induced saline current. The ultrasonic transducer was lowered in from the top of the tube. Thus, this geometry coaxially aligned the directions of the B field and sound detection. This coil topology was efficient at inducing large currents through the saline and phantom, but it suffered from nonlinear harmonic production in the ferrite rings. Ferrite heating also became a problem with extended testing times.

##### Coil 6: Oval solenoid

**Fig. 6:**
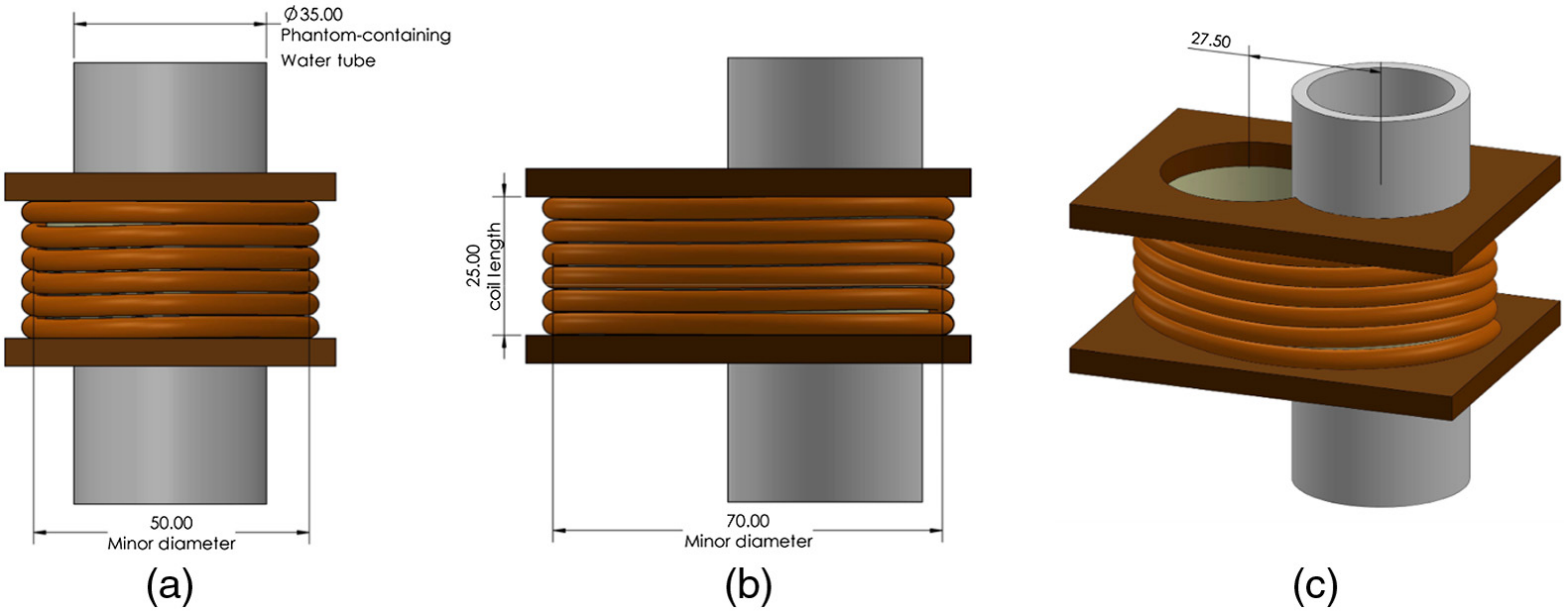
Oval solenoid with off-center phantom positioning from (a) side, (b) front, and (c) isometric views. Off-center positioning avoids nulls in the induced E field. Units of measurement are millimeters.

A single 6-turn oval solenoid coil (25 mm length) was constructed as shown in Fig. 6. The phantom-containing water tube (35 mm diameter) was positioned off-center to avoid the symmetry that nulls the B-induced E field in the center of a circular coil. A phantom placed in the tube will be surrounded by roughly 1/2 of the turn circumference with the other half some distance away. The coil is 5-1/5 turns of high-voltage wire wound around a binocular core and 25 mm in length. The two holes in the wood coil form were made by a 3.5-cm drill on 27.5 mm centers. Wall thickness was about 2.5 mm. The major diameter of the coil was 70 mm, the minor diameter was 50 mm, and the length was 25 mm.

#### 2.1.3 Transducers

US was detected using an Olympus A311S focused transducer with a resonant frequency of 10 MHz, a focal length of 5.1 cm, and a diameter of either 1.3 or 1.9 cm.

### 2.2 Image Acquisition and Processing

Signal averaging, coherent processing with amplitude envelope display, active damping of the transmit burst, or a combination thereof was performed.

Coherent detection was implemented to provide the highest possible SNR by rejecting all nonphase coherent noise frequencies. A 40-MHz master crystal oscillator was used as the time base. This clock was divided by four with a circuit that produced two 10-MHz clock references with an exact 90-deg phase relationship (I and Q). The I signal was further divided by two to make the phase-locked 5-MHz transmitter burst. The output from the 10-MHz transducer was amplified with a very low noise amplifier and then processed in a Costas loop referenced to the I and Q 10 MHz clocks. Outputs from the I and Q channels were summed in an analog circuit that performed the function $\sqrt{(I^{2}\;+\;Q^{2})\;}$, then filtered to produce the scalar amplitude of the received pulse.

Single point data were displayed on the oscilloscope with time of flight (TOF) defining the region of interest generating the EMAI signal. The oscilloscope used was an Agilent DSO5034A which had the capability to signal average. Depending on the strength of the returned signal, signal averages ranging from 2 to 32,768 acquisitions could be performed. The oscilloscope was synchronized with a pulse produced by the coherent signal processor. 2-D scans were obtained with a single transducer by rastering across the phantom with a 2-D mechanical stage. Signal data were processed in Mathematica for 2-D images.

To maximize transmitter efficiency, the coils were resonated at 5 MHz with as high a circuit Q as possible commensurate with desired ring-up time. To quench ringing in the tuned circuit at the completion of the transmit burst, active damping was used. At the end of the desired transmit burst (typically 5 to 20 cycles or 1 to 4  μs) coil excitation continued but with reversed phase drive until all the energy stored in the coil circuit was extracted. This technique produced well-behaved transmit pulses with very fast ring down times.

### 2.3 Phantoms

Room temperature vulcanizing (RTV) silicone rubber phantoms in both silver and nickel formulations were used in a bath of either water or saline. Small RTV spheres were suspended by nonconductive rods. An emblem of RTC silicone was constructed as described below to demonstrate resolution and image contrast in a 2-D image. A simple phantom consisting of the interface between saline and air was used to track TOF and EMAI signal intensity as a function of salinity (and thereby conductivity). Tissue phantoms were untreated beef and chicken liver parts.

## 3 Results

### 3.1 EMAI from Conductive Silicone

**Fig. 7:**
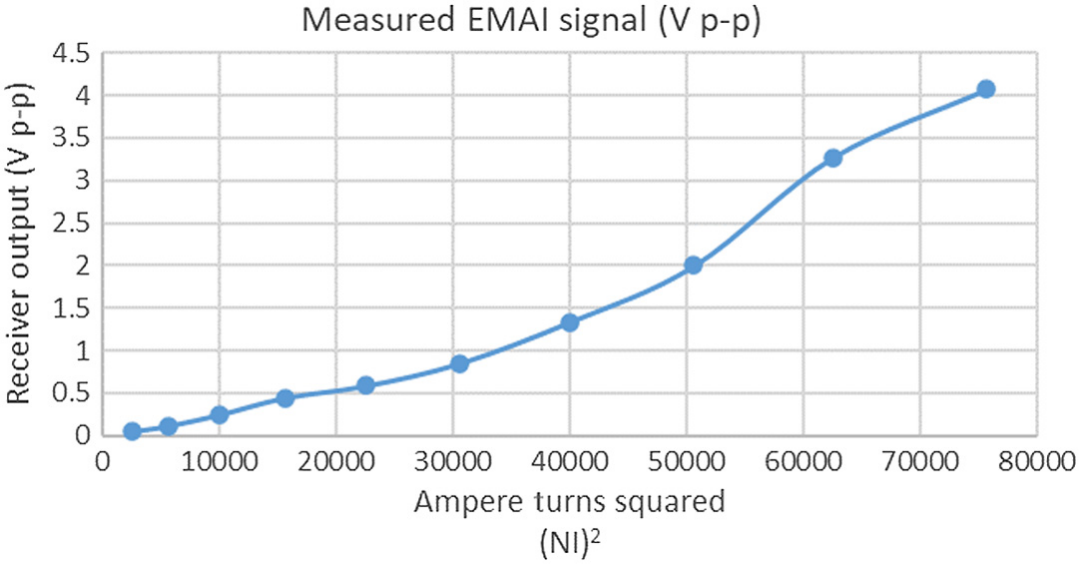
Measured 10-MHz EMAI signal in volts, peak-to-peak as a function of A-T for 20-turn Helmholtz coils.

Figure 7 shows a plot of the 10-MHz EMAI values obtained from conductive RTV silicone suspended in water pulsed with 5-MHz RF delivered by coil 1, the 20-turn, 76-mm, Helmholtz pair. As predicted by Eq. (2), EMAI signal intensity varies approximately linearly with $(\mathrm{NI}{)}^{2}$. Coil current was determined by measuring the voltage across the resistor in series with the coil used to set the Q of the tuned circuit affording a high degree of linearity in the coil setup. Departures from linearity in the acquired data can be attributed to amplitude variations arising from differences in acoustic coupling with the phantom and to interference effects. The sound field from one part of the phantom is coherent with those from neighboring regions. The sound field at a distant point is the vector sum of individual contributions and shows significant spatial variability (speckle). It is possible some of the variability in the plot stems from point-to-point measurement uncertainty incurred over the time span of the measurement.

### 3.2 Ferrite Loaded Coils

While the ferrite core as described above for coil 2 was intended to provide flux enhancement through the phantom volume, the increase in flux was largely restricted to the coil core rather than through the gap. Therefore, the boost in induced field in the phantom was minimal. Further, the nonlinearities associated with using ferrites at high flux levels created harmonic production, which distorted the coil waveform thereby complicating the 1f-2f methodology used with EMAI. In addition, with long mechanical scans, ferrite heating exceeded tolerable limits.

### 3.3 EMAI from Excised Tissue Phantoms

Various phantoms were selected to approximate the acoustic properties of *in-vivo* tissue along with the expected spatial variability of conductivity encountered in the body. Excised tissues rather than inorganic phantoms were used to more closely simulate *in-vivo* conditions. Example results from chicken liver and beef liver are shown below to further demonstrate feasibility.

**Fig. 8:**
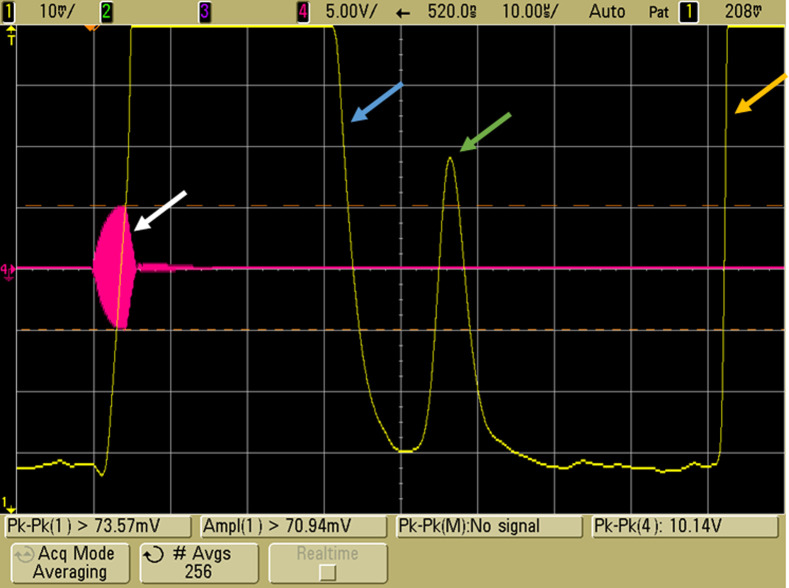
EMAI signal from a saline–air interface. The 4-μs RF burst with active damping is noted by the white arrow. The yellow tracing is the coherent amplitude envelope received by the transducer. Overload from the transmit burst is marked by the blue arrow. The EMAI signal (green arrow) resulting from the conductivity change at the interface is seen at ∼45  μs representing one TOF. The large peak (yellow arrow) at 2 TOF is the pulse echo resulting from RF leakage into the transducer.

**Fig. 9:**
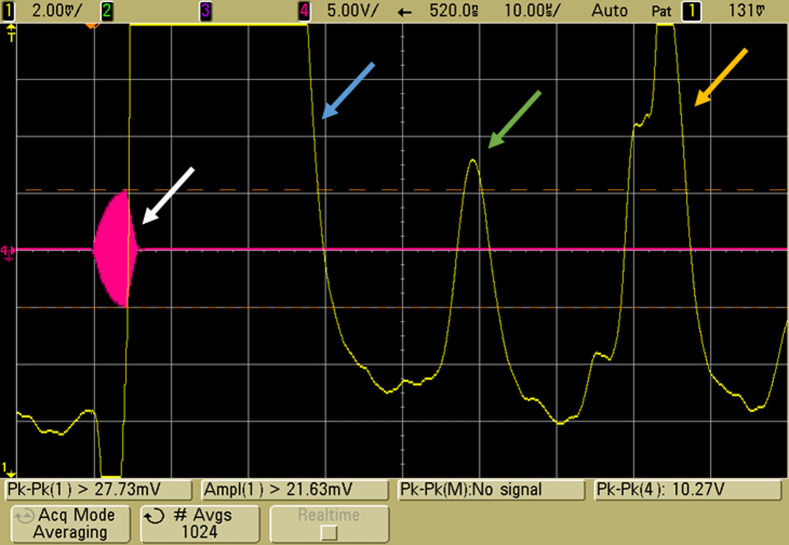
EMAI signal from a section of chicken liver suspended in saline. The pink tracing displays the 4-μs RF burst with active damping (white arrow). The yellow trace is coherent processing amplitude envelope from the receiver showing effects of transmit burst (blue arrow), EMAI signal from tissue/saline conductivity gradient (green arrow) at one TOF and the 2 TOF pulse echo (yellow arrow) resulting from excitation of the transducer by the RF burst.

**Fig. 10:**
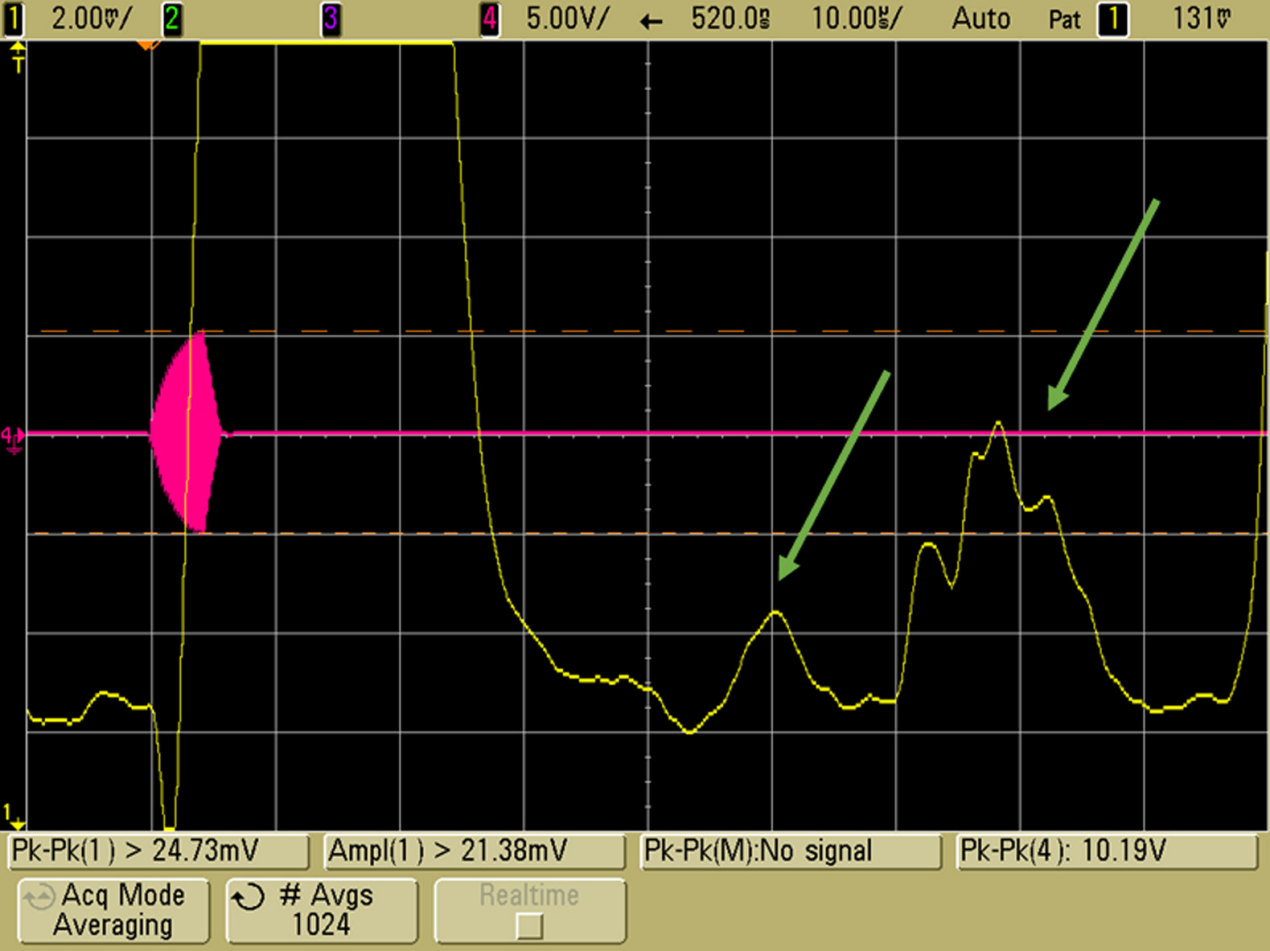
EMAI signal from a section of beef liver suspended in saline. The pink tracing displays the 4-μs RF burst with active damping. The yellow trace is coherent processing amplitude envelope from the receiver. Green arrows indicate the position of the two macroscopically distinguishable sections of liver including vessel stubs.

EMAI signals demonstrated in Figs. 8–10 were obtained with coil 3, the modified Helmholtz coil described in Sec. 2.1.2, subsection “Coil 3: Modified Helmholtz coils,” a 10-MHz transducer in a saline water bath, the burst transmitter operating at 5 MHz with 2000 to 3100 A-T pk-pk, and 4  μs pulses on a repetition rate of 1 ms. Coherent processing with signal averaging ranging from 256 to 1024 acquisitions was used. In each figure, the pink tracing represents the RF burst, and the yellow tracing represents the amplitude envelope output from the transducer. The initial large peak is due to harmonic excitation of the transducer at the time of the RF burst, and the large peak on the right is a 2-TOF pulse echo from that burst. In the center, at one TOF depending on phantom placement, is the 10-MHz EMAI signal generated by the RF-induced E fields.

For reference, Fig. 8 displays the EMAI signal obtained from a saline–air interface. The tank positioned in the coils was filled with saline (9 g/L NaCl) to a height that placed the liquid–air interface just beyond the focal point of the transducer. The conductivity gradient from the interface resulted in an EMAI signal at one TOF shown in the figure at the green arrow. Signals were averaged with 256 acquisitions resulting in a 10-mV signal. Varied interface positioning tracked with TOF (not shown).

In Fig. 9, the EMAI signal from a 1-cm piece of macroscopically homogenous chicken liver is displayed. The SNR was predictably lower than for a saline–air interface. With signal averaging of 1024 acquisitions, the peak amplitude was ∼9  mV.

The feasibility of detecting tissue architecture was tested with a heterogenous piece of beef liver that included vessel stubs. In Fig. 10, multiple EMAI returns are seen at TOF values representing the respective sections of the phantom. The overall SNR was lower than in the chicken liver such that the same signal averaging (1024 acquisitions) resulted in amplitude peaks of 2 to 6 mV with the higher values obtained from the section including vessels.

Axial resolution is a function of the wavelength of the US and the pulse width. Pulse width is a function of the number of cycles used in the RF burst and the Q of the coil circuit. The drive pulse is seen in the red trace of Fig. 10. The number of cycles in the drive was the minimum required for pulse envelope to reach maximum value and this occurred in 2  μs. Active damping quenched the pulse such that the overall pulse width was 3  μs. Reading the received peak pulse amplitude with a precision of tenths of a microsecond gives axial resolution on the order of 0.5 mm.

Lateral resolution was set largely by the focal waist of the focused transducer and axial phantom location. For the 10-MHz US frequency and 50-mm focal length transducer, lateral resolution is on the order of few wavelengths or <0.5  mm.

With the use of coil 6, the off-center solenoid, avoiding the central B-field null improved EMAI detection such that, in addition to excised liver, signals were obtained from other phantoms including adipose in saline, which with other coil configurations was undetectable with 2024 A-T p-p. Signal peaks of 130 mV were obtained with signal averaging of 2048 acquisitions.

### 3.4 Experimental Results Compared to Theoretical Predictions with the Modified Series Connected Helmholtz

The theoretical prediction of the EMAI signal intensity was compared to that obtained experimentally using the series-connected modified Helmholtz coil (coil 4) described in Sec. 2.1.2, subsection “Coil 4: Solenoid.” A specific coil configuration was used for analysis to support agreement between theory and experimental results which is likely generalizable. In comparison to coil 3, the electric field at the radius of the loops can be shown to be improved by a factor of 2.17 (6500 versus 3000 V/m at NI = 1600 A-T). The resulting EMAI signal intensity is thus expected to improve by a factor of $2.17^{2}$ or 4.7. With conductive RTV in water, the skin depth is small compared to phantom dimensions, and thus the stress on the phantom, $S_{\mathrm{RF}}$, given as

$$S_{\mathrm{RF}}=B^{2}/2\;\mu =2.4\;{N/m}^{2},$$ (3)

is primarily due to the magnetic field, using $B=2.45\times 10^{-3}\;\mathrm{Wb}/m^{2}$. The EMAI pressure is given as

$$P_{\mathrm{EMAI}}=\frac{Z_{\mathrm{water}}}{Z_{\mathrm{water}}+Z_{\mathrm{RTV}}}S_{\mathrm{RF}},$$ (4)

with $Z_{\mathrm{water}}=1.5\times 10^{6}\;\mathrm{kg}/m^{2}\;s$ and $Z_{\mathrm{RTV}}=1.1\times 10^{6}\;\mathrm{kg}/m^{2}s$, the resulting EMAI pressure is $P_{\mathrm{EMAI}}=1.38\;N/m^{2}$. For a transducer of 1.27 cm diameter with a 5.08-cm focal length, $V_{\mathrm{transducer}}=0.7S_{\mathrm{RF}}=0.97\;\mu V$. The cumulative gain in the analog signal processing chain was 67,320 giving an expected output voltage of $V_{\mathrm{output}}=67\;\mathrm{mV}$, which correlated well with the experimental measurement of 62 mV obtained by dividing the signal level at the transducer output displayed on the oscilloscope by the overall amplifier gain.

### 3.5 Two-Dimensional Image Demonstration of Resolution and Image Contrast

**Fig. 11:**
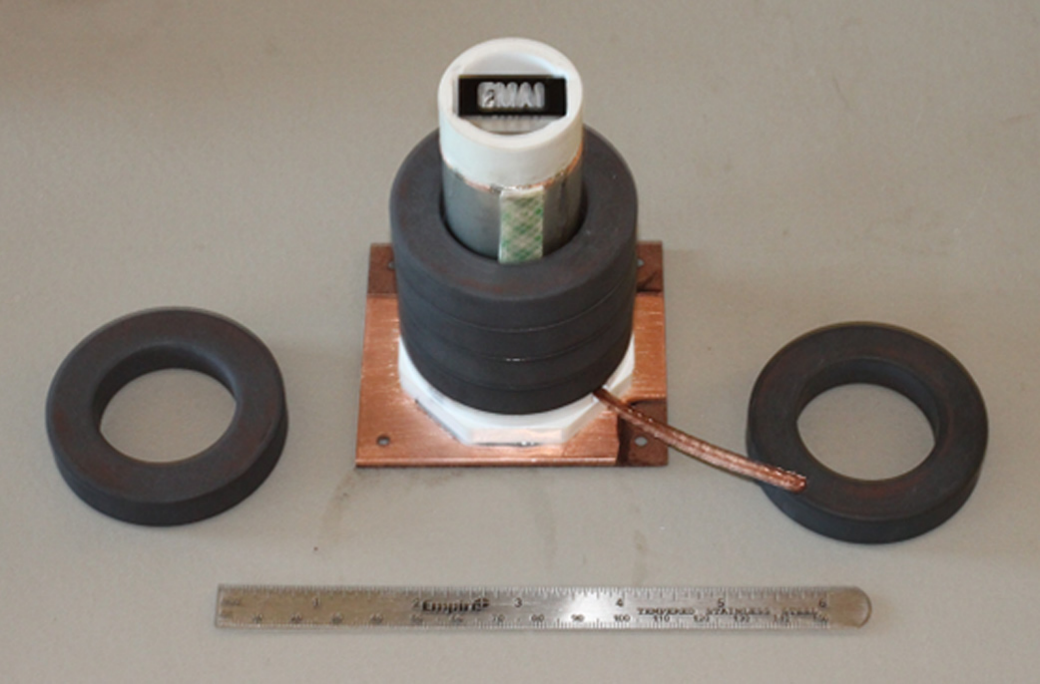
Positioning of the “EMAI” emblem in the toroid coil assembly diagrammed in Fig. 5.

**Fig. 12:**
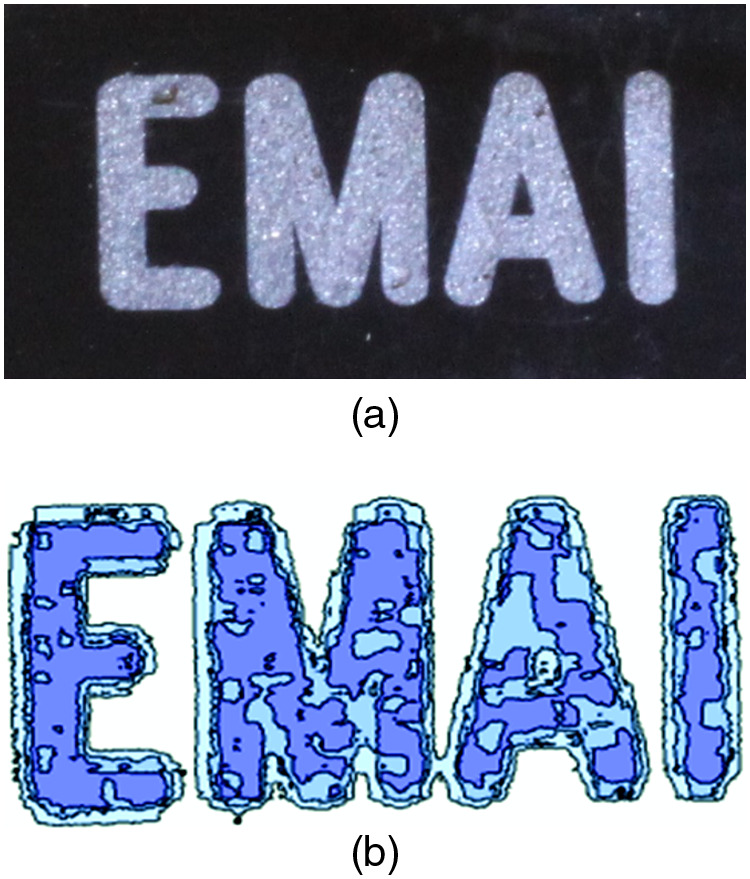
Comparison of EMAI image to optical image of a conductive silicone RTV phantom: (a) optical image of an engraved plastic plate 12.7  mm×2.5  mm with letter height 7.1 mm, etched in grooves 3.2 mm in depth which were filled with conductive silicone; (b) EMAI image of same plate using 5-MHz RF delivered by a modified toroid coil (described in Sec. 2.1.2, subsection “Coil 5: Toroid with ferrite sleeves”) providing 2500 A-T in 3  μs pulses at a 1-ms repetition rate. The image signal is 10 MHz US with darker blue representing higher signal intensity. Graininess in the image is speckle caused by coherence in the US resulting from monochromatic magnetic excitation.

To demonstrate resolution and image contrast, a 2-D EMAI image of a phantom (positioned in coil 5 as shown in Fig. 11) is displayed next to the corresponding optical image in Fig. 12. The phantom consisted of a plastic plate with overall dimensions of 12.7  mm×2.5  mm in which letters were etched in grooves 3.2 mm in depth and filled with conductive RTV silicone. The assembly was pulsed with 5-MHz RF, 2500 A-T, in 3μs pulses at a 1-ms repetition rate. Mathematica was used to display the data from the coherently processed signals into the 2-D image shown. In the EMAI image, darker blue corresponds to higher signal intensity. Spatial resolution was sufficient to show the EMAI signal drop in the triangular space in the letter A which measures 1.2  mm×0.6  mm. Graininess in the image results from speckle caused by coherence in the US due to monochromatic magnetic excitation.

### 3.6 Summary of Findings Across Coil Configurations

A brief summary of the design intent and findings described above for each of the coil configurations is provided in Table 1.

**Table 1:** Summary of findings in different RF coil configurations.

Coil configuration	Design intent	Results
Helmholtz (subsection “Coil 1: 20-turn, 76-mm Helmholtz coils”)	Verify SNR as a function of NI.	EMAI signal intensity varies as (NI).^2^
Helmholtz with ferrite core (subsection “Coil 2: Helmholtz coils with ferrite rods”)	Flux enhancement to raise effective NI.	Minimal increase in induced field in phantom. Problems with nonlinearities and heating.
Modified Helmholtz (subsection “Coil 3: Modified Helmholtz coils”)	Maximize B, E fields experienced by the phantom.	EMAI signals demonstrated feasibility in excised tissue with coherent processing.
Solenoid (subsection “Coil 4: Solenoid”)	Investigate circumferential current, with coaxial US and B field.	Experimental agreement with theoretical prediction of EMAI signal strength.
Transformer (subsection “Coil 5: Toroid with ferrite sleeves”)	Maximize B and E field coupling to saline column.	2-D image demonstrating resolution, image contrast in conductive phantom.
Oval solenoid (subsection “Coil 6: Oval solenoid”)	Off-center phantom placement to avoid field nulls induced by symmetry.	Improved SNR in excised tissue allowed detection of adipose in saline.

Note: Subsections listed in the “Coil configuration” column refer to Sec. 2.1.2.

## 4 Conclusions

EMAI can be an important complement to other medical imaging modalities because it uses high-resolution US imaging to display tissue electrical conductivity gradients. The electrical conductivity gradients reveal disease states primarily through the difference in diseased and normal tissue vascularity.

The experiments with excised tissue phantoms reported in this paper have demonstrated that acceptable signal to noise EMAI signals can be obtained with good resolution and image contrast. As expected, they showed that the occurrence of the US signal at twice the RF excitation frequency was important in obtaining good signal to noise.

This work has also demonstrated that RF induction coils with practical pulsed power sources are an effective noncontact means of obtaining the electric fields that generate the imaging US. When coils are used, the largest EMAI signals are obtained from the portions of the phantoms located closest to the coil radius, as that is where the induced electric fields are the largest. (Although not described here, we have also demonstrated that good signal to noise EMAI can also be obtained by introducing the electric fields by direct contact electrodes.) In coil 5, the B-induced-E, the E field between the ends of the “coil” (actually a linear conductive sleeve), and the direction of US propagation were all axially aligned. This coil configuration produced optimum EMAI signals; however, further work is required to verify causality.

The signals and images in the experiments were all obtained with by scanning a single piezoelectric detector resonant with the US frequency. Improved signal to noise can be obtained and the data collection time considerably reduced if linear and 2-D detector arrays are employed instead of a single detector.
